# Failure or progress?: The current state of the professionalisation of midwifery in Europe

**DOI:** 10.18332/ejm/115038

**Published:** 2019-12-17

**Authors:** Joeri Vermeulen, Ans Luyben, Rhona O’Connell, Patricia Gillen, Ramon Escuriet, Valerie Fleming

**Affiliations:** 1 Department of Health Care, Knowledge Centre Brussels Integrated Care, Erasmus Brussels University of Applied Sciences and Arts, Brussels, Belgium; 2 Centre for Midwifery, Maternal and Perinatal Health, Faculty of Health & Social Sciences, Bournemouth University, Bournemouth, United Kingdom; 3 Department of Health Services Research, University of Liverpool, Liverpool, United Kingdom; 4 Cantonal Hospital Graubünden, Chur, Switzerland; 5 School of Nursing and Midwifery, University College Cork, Cork, Ireland; 6 Southern Health and Social Care Trust, Northern Ireland, United Kingdom; 7 Institute of Nursing and Health Research, Ulster University, Northern Ireland, United Kingdom; 8 Catalan Health Service, Barcelona, Spain; 9 National Commission of Midwives, Ministry of Health, Barcelona, Spain; 10 School of Nursing and Allied Health, Liverpool John Moores University, Liverpool, United Kingdom

**Keywords:** education, midwives, profession, health care, midwifery, maternity care

## Abstract

**INTRODUCTION:**

Throughout Europe midwives called for increasing professionalisation of midwifery during the 1980s and 1990s. While the Bologna Declaration, in 1999, supported this development in education and research, it remains unclear how other fields, such as practice, have fared so far. This study therefore aimed to explore the current state of professionalisation of midwifery in Europe.

**METHODS:**

An exploratory inquiry was conducted with an on-line semi-structured questionnaire. Its content was based on the Greenwood sociological criteria for a profession. Descriptive statistics and thematic content analysis were used to analyse the data. Participants were national delegates from member countries to the European Midwives Association.

**RESULTS:**

Delegates from 29 European countries took part. In most countries, progress towards professionalisation of midwifery has been made through the move of education into higher education, coupled with opportunities for postgraduate education and research. Lack of progress was noted, in particular in regard to midwifery practice, regulation, and leadership in health care provision and education. Most countries had a code of ethics for midwives as well as a midwifery association. Based on organisational collaborations with other disciplines, the sustainability of a distinct professional culture was unclear. An increased focus on future development of midwifery practice was proposed.

**CONCLUSIONS:**

Progress in midwifery education and research has taken place. However, midwives’ current roles in practice as well as leadership and their influence on healthcare culture and politics are matters of concern. Future efforts for advancing professionalisation in Europe should focus on the challenges in these areas.

## INTRODUCTION

During the 1980s and 1990s, in order to professionalise midwifery, midwives throughout Europe called for changes to their professional bodies. In particular, midwives were concerned about issues of autonomy and control^1,2^. They were not alone in this quest as increased professionalisation was a central aim of multiple healthcare provider groups at that time^3,4^. Such groups were often referred to as ‘callings’, ‘charity’, ‘occupations’ and ‘semi-professions’^5,6^, with their work based on applied knowledge from more established academic disciplines such as medicine.

While there are many definitions of professionalisation, it is generally understood as ‘the process by which an occupation develops the characteristics of a profession’^7^. This also might involve an increased differentiation of professional structures^8^. Greenwood’s taxonomy distinguishes between an occupation and a profession, which remains valid today^2,4^. The defining differences include: own body of knowledge, recognised authority, broader community sanctions, own code of ethics, and professional culture sustained by formal professional associations. Increasing professionalisation of midwifery thus should lead to strengthening of its professional autonomy, and so increase its recognition, prestige and income, as well as political influence. In line with the drive for professionalisation, it was expected that professional associations would play a central role in controlling their own profession, including defining and accrediting education programmes, (re)certification of its own professionals, developing best practice standards, and sanctions to be taken in litigation processes concerning midwifery^2,9^. In essence, professionalisation should enable midwifery to regain a partnership in designing and changing healthcare, at the same level as other health professionals.

Whereas Europe has the longest global tradition in midwifery, it also has greatly diverse political and healthcare systems^10^. Despite this, European unity has been a key aim since the end of World War II with the common market, founded in 1957, paving its way. In 1980, as these expanded, health services came under scrutiny, the EU Directives (EEC/80/154 and EEC/80/155; now 2013/55/EU)^11^ were developed, which aimed at promoting freedom of movement of midwives throughout Europe. The directives included common guidelines in both practice and education, for all member countries involved (European Parliament and the Council of the European Union 2013). Building on these, several countries started to move midwifery education from vocational into higher education as part of the journey towards professionalisation.

Since 1999, through its drive for common systems in the Higher Education sector, the Bologna Declaration has supported the professionalisation of midwifery in Europe^12,13^. With a strong focus on evidence-based theory and the encouragement of critical thinking, the move into higher education aims to equip midwives with increased evidence-based practice and thus to meet the demands of modern maternity care^14^.

In many countries, the principles of the Bologna process have been realised over the last decades, although to varying degrees throughout Europe^15^. Many midwifery programmes are currently provided at Bachelor’s level in some countries, however midwifery education is still offered as a vocational or an apprenticeship model^16,17^. The provision of higher degrees and continuous professional development also vary^18,19^, particularly in continental Europe.

As a consequence of the move into higher education, opportunities for midwives to become active in research, and thus create their own body of knowledge, have increased. The authors of ‘Having a baby in Europe’^20^ urged midwives to undertake research to study and underpin midwifery practice. Since that time, midwifery research has increased. Critics, however, have pointed out that midwifery research still lacks a unique underpinning philosophy necessary for creating its own body of knowledge^21^. In the English-speaking countries, the development of midwifery research was initiated during the 1980s and 1990s^22^. The uptake of research by midwives in the majority of continental Europe, however, only happened upon the implementation of the Bologna Declaration^23,24^.

In contrast to the apparent progress in education and research, at the same time the economic and societal changes affected healthcare in general also affected maternity care and thus the practice of midwifery. These changes in particular include cost-saving changes, such as reduced staffing and integration of care, as well as increased focus on economics, digitalisation and medicalisation of care^25,26^. For some countries, studies have discussed their effects and consequences, in particular with regard to values and culture^27-30^. It has been hypothesised that these changes might be a threat to the autonomy of midwives, the transfer and implementation of midwifery philosophy and competencies, and, in summary, result in a backlash to the endeavour of professionalisation of midwifery^9,31^. For the majority of European countries, however, knowledge about the effects of these changes is lacking.

In order to evaluate progress and to be able to focus on priorities for future action, this study aimed to explore the current state of professionalisation of midwifery in Europe.

## METHODS

### Design

An exploratory research design was used to achieve the aims of this study. New knowledge gained should serve as a foundation for explanatory studies and more in-depth investigations^32^. Thus, an exploratory inquiry using an online questionnaire was designed and conducted.

### Participants and setting

Key information on midwifery for each European country was provided by delegates of the European Midwives Association (EMA). EMA has an important role in securing quality midwifery education and practice throughout Europe^15^, and currently incorporates 37 midwifery organisations from 30 countries. The countries are: 26 from the 28 EU countries, two from the European Economic Area (Iceland and Norway) and two from the Council of Europe (Switzerland and Turkey).

While delegates from national midwifery associations were chosen for representation in EMA due to relevant experience in the field, they were considered experts on midwifery in their country. The study was approved by EMA’s Executive Board in May 2019. Access to the participants was requested through EMA and subsequently from each member country’s national midwifery association. Informed consent from the participants was gained through their agreement to participate on the first page of the inquiry, with completion of the inquiry also indicating their willingness to take part. The study was self-funded. Additional ethical approval was not required.

### Data collection

A semi-structured questionnaire, comprising both open and closed questions, was constructed in English, in April and May 2019. Its structure was based on the Greenwood sociological criteria for a profession. Each closed question was followed with an option to comment in more detail. The content was based on existing studies and policy documents. Once developed, the questionnaire was extensively discussed and adapted within the study team. Upon final agreement, transfer into a survey format on the platform SurveyMonkey^®^ (SVKM) took place. In order to test the content and international understanding, a small pilot study with participants in four countries (Spain, Belgium, Ireland, Switzerland) was conducted before its ultimate launch.

The final questionnaire included 56 questions in five sections: 1) A unique body of knowledge (20 questions), 2) Authority recognised by its clientele (19 questions), 3) Broader community sanctions (11 questions), 4) A code of ethics (3 questions), and 5) A professional culture sustained by a formal professional organisation (3 questions). In addition, two open questions about past and anticipated changes in midwifery were posed. Each closed question was followed by an option to comment. Participants were asked to provide contact data, in case clarification about their responses was needed.

In early June 2019, 37 delegates from 30 countries were invited by e-mail to participate in the inquiry. The invitation included information about the study, an informed consent form and a link to the survey site. If the initial participant did not answer, a second person from the same organisation was invited. A reminder was sent at three and five weeks after the initial invitation. The survey was closed on July 15th 2019.

### Data analysis

The responses of the closed questions were analysed using descriptive statistics, such as absolute values and percentages. Being of an exploratory nature, the survey did not aim for generalisability. Thematic content analysis was used to analyse the answers to the open questions^33^. The emerging categories were compared, reflected upon and agreed to, within the study team.

## RESULTS

Thirty-five delegates from 29 European countries participated in this study (Table 1). For six countries multiple or incomplete answers were received. In these cases, the delegate concerned was contacted for clarification of the data and the answers were collated as one. No responses were received from Lithuania. The resulting data of 29 European countries are reported in this study, using Greenwood criteria as a structure.

**Table 1:** Participating associations

*Country*	*Midwifery Association*
Austria	Austrian Midwives Association
Belgium	Belgian Midwives Association
Bulgaria	Alliance of Bulgarian Midwives
Croatia	Croatian Chamber of Midwives
Cyprus	Cyprus Nurses and Midwives Association, Midwife Committee
Czech Republic	Unie porodních asistentek
Denmark	Danish Midwives Association
Estonia	Estonian Midwives Association
Finland	The Federation of Finnish Midwives
France	Collège National des Sages-Femmes de France
Germany	DHV e.V. Deutscher Hebammenverband
Greece	Hellenic Midwives Association
Iceland	Ljósmæðrafélag Íslands
Ireland	Irish Nurses and Midwives Organisation. Midwives’ Section
Italy	Societa ltaliana di Scienze · Ostetrico-Ginecologiche e Neonatali
Latvia	Latvian Midwives Association
Luxembourg	Association Luxembourgeoise des Sages-Femmes
Malta	Malta Union of Midwives and Nurses
the Netherlands	Royal Dutch Organisation of Midwives
Norway	Norwegian Association of Midwives
Portugal	Associação Portugues de Enfermeiros Obstetras
Romania	Independent Midwives Association
Slovakia	Slovak Chamber of Nurses and Midwives
Slovenia	Nurses and Midwives Association of Slovenia
Spain	Federación de Asociaciones de Matronas de España
Sweden	Swedish Association of Midwives
Switzerland	Schweizerischer Hebammenverband/Fédération suisse des sages-femmes
Turkey	Ebeler Dernegi
UK	Royal College of Midwives

### A unique body of knowledge

The first section of the questionnaire focused on the acquisition of midwifery knowledge, specifically education and research. In 26 countries basic midwifery education took place at a university; in 19 at Bachelor’s and in seven at Master’s level (Table 2). Access to a Master’s programme in midwifery usually required a Bachelor’s of nursing, except for example in France and Greece. In four countries, initial midwifery was still offered as a vocational programme at a Diploma level. While being in transition, in Germany both vocational and Bachelor’s programmes were provided. In 23 countries, direct entry midwifery was provided with a programme length that ranged from three to four and half years. Post nursing midwifery programmes were available in 14 of the 29 countries. Most of these (n=9) were of two years duration. The majority of the participants indicated that the head of the midwifery programme was a midwife (n=22). When not, most often it was a doctor, but also may have been a nurse. Most (n=25) indicated that the EU Directives had had an impact on midwifery education.

**Table 2:** Outcome level of midwifery education

*Country (n=30)*	*Bachelor (n=19)*	*Master (n=7)*	*Diploma (n=4)*	*Missing data (n=1)*
Austria	X			
Belgium	X			
Bulgaria	X			
Croatia			X	
Cyprus		X		
Czech Republic	X			
Denmark	X			
Estonia			X	
Finland	X			
France		X		
Germany	X		X	
Greece		X		
Iceland		X		
Ireland	X			
Italy	X			
Latvia	X			
Lithuania				X
Luxembourg			X	
Malta	X			
Netherlands	X			
Norway		X		
Portugal		X		
Romania	X			
Slovakia	X			
Slovenia	X			
Spain		X		
Sweden	X			
Switzerland	X			
Turkey	X			
UK	X			

In all countries, except Luxembourg, opportunities for midwives to do postgraduate education were available, such as a Master’s and a PhD in midwifery or in other disciplines. The complexity of provision across Europe was also highlighted, with five countries indicating that they offered a Master’s in midwifery, but a PhD only in other subject areas, while in one country it was possible to undertake postgraduate study in nursing, but not midwifery. Of the 28 countries where postgraduate study was available, 18 had Master’s and PhD studies supervised by midwives. In the absence of midwives as supervisors, this role was taken over by other health professionals including doctors, nurses and allied health professionals.

In many countries, midwives had opportunities to undertake research but not in Bulgaria, Luxembourg and Romania. In most countries, research was part of higher education courses. Midwives in 25 countries could apply for funding, with most funding coming from the university sector. Most participants had limited knowledge of their involvement in its allocation, but it was clear that in some countries, such as Denmark and Spain, there was active lobbying for more involvement in the future. A growth in the area of midwifery research since 1999 was noticed. The number of professors of midwifery was recognised as a big change, as was the fact that midwives were now leading research projects and midwifery research institutes had been established in some countries.

All countries indicated that there was some continuous professional development (CPD) available for midwives. In some counties, CPD was regulated, such as Belgium and the UK, in which CPD is mandatory with a minimum number of hours. Funding varied according to place of work in a country. In 21 countries midwives retained their right to practise throughout their career, although revalidation or re-accreditation was required in 10 countries every three to five years.

### Authority recognised by its clientele

The second section of the questionnaire focused on professional autonomy and midwifery practice. Women were able to access maternity care through an obstetrician, General Practitioner (GP) or midwife, with the majority accessing care through an obstetrician (n=21). Where private maternity care was available, this was always with an obstetrician (n=24) (Table 3). Some countries (n=11) had independent midwives whom women could access directly. For care during pregnancy, the obstetrician was often the main care provider, although the GP and midwife could be involved. One country reported that the public health nurse provided the majority of antenatal care (Finland), and another (Malta) that this care was provided by GPs and obstetricians only.

**Table 3:** Access to the national maternity care system and main care providers in antenatal care* (N=29)

*Access to the system*	*Private*	*Public*
Obstetrician/Gynaecologist	24	21
General Practitioner (GP)	3	9
Midwife	7	14
Not applicable	3	
***Main care provider in antenatal care***	***Private***	***Public***
Obstetrician/Gynaecologist	22	21
General Practitioner (GP)	2	8
Midwife	9	15
Not applicable or other	4	1

* Multiple answers were possible.

Many participants commented that midwives’ practice in their country was in line with the activities of the EU Directives, but that they worked in an obstetrician-led system of maternity care. Nine participants stated that they could not work according to International Confederation of Midwives (ICM) competencies and EU Directives. In many countries (n=25), nurses were involved in providing aspects of care to low-risk women. Mostly, this was in postnatal care (n=19) but also in antenatal (n=6) and intrapartum care (n=5), taking up midwifery roles. A shortage of midwives was cited as a reason for this.

The degree of autonomy for midwives in providing care to women in labour varied. In seven countries, midwives reported that most could not provide autonomous care for low-risk women in labour, though this varied throughout the country. In some countries, midwives had to call a doctor for the birth. Midwives working in hospitals had limited control over work conditions, e.g. shifts worked or the provision of one-to-one care. In 20 countries, advanced roles, such as in lactation, ultrasound (n=4) and bereavement and pregnancy loss (n=5) were available, in some countries these were related to an increase in salary. Most often a CPD, sometimes a Master’s qualification (e.g. consultant midwife or professional midwifery advocate, UK) was required. In most hospitals, heads of the maternity departments were midwives (n=17); others stated that a doctor or a nurse was in-charge.

Countries that offered midwifery-led services or independent midwifery reported greater autonomy for these midwives. Opportunities to provide continuity of care were reported in 16 countries. There were birth centres in 15 countries, and in 18 countries midwives could work as independent practitioners. Professional indemnity insurance was cited as a problem for midwives working outside of the hospital system. In seven countries, it was illegal for midwives to provide homebirth.

In relation to changes in midwifery autonomy and practice since 1999, 19 delegates experienced and perceived an increase in the medicalisation of birth, and considered reductions in their scope of practice as threats. Delegates also cited opportunities for some midwives such as the development of birth centres and midwifery-led care, as well as an increase in educational opportunities and advanced or specialist roles.

### Broader community sanctions

Legislation protected the midwifery profession in most countries. Except in Slovenia and Romania, national health policy determined midwives’ scope of practice. Regulation for midwives was reported for only seven countries, and joint regulation for both midwives and nurses in six countries. Delegates from 21 countries reported that midwives’ legal scope of practice corresponds with EU Directives. All participating countries did require a registration for practising midwifery, but only a few required revalidation.

The national government was responsible for regulating midwifery practice in 15 countries, through Ministries of Health or Education. In nine countries, this responsibility for practice lay with midwifery associations or special councils. Some of these also regulated nursing, for example in Cyprus and the UK. In 24% of the countries, a midwife held a regulating position in the Department of Health. Some countries reported that, in general, a nursing director took up this role. Only three countries mentioned a midwife in the Department of Education.

### A code of ethics

The Czech Republic, Luxembourg and Spain stated that they had no code of ethics for midwives but the remaining 26 countries answered positively. Eighteen countries had a code of ethics uniquely for midwives, seven of which were based on the ICM code of ethics. The remaining eight countries with codes of ethics shared these with other professions, mainly nurses, although the UK, Romania and Greece reported having a common code for all health professionals. In France, the midwives’ code is based on that available for doctors.

### A professional culture sustained by a formal professional organisation

In all 29 European countries, a professional organisation unique for midwives was identified, and seven countries had more than one association for midwives, sometimes combined with nurses or other health care professionals. Seven countries stated they had a midwifery unit under the auspices of a larger nursing organisation. The midwifery associations in Bulgaria and Romania lacked legal recognition. Eleven delegates believed that their association had little or indirect influence on the policy-making of the national government; five (Cyprus, France, Germany, the Netherlands and Turkey) believed they had a significant political impact.

### Changes in midwifery since 1999

Both the EU Directives and the Bologna process coupled with changes in midwifery education were viewed as having a significant impact on midwifery education and practice in most countries (n=18). Several countries reported the move to higher education, and the opportunity to become research-active as a profession, as an important trigger for these changes. Seven countries attributed these changes to the midwives themselves. In some European countries, political causes were mentioned as drivers including changes in the political system, as well as health care politics and, as a consequence, midwives’ roles.

### Anticipated future changes in midwifery

In general, participants were positive in regard to anticipated changes in midwifery (Table 4). Most delegates reported further development of midwifery practice as an important future issue. Developing midwifery practice was understood as implementing strategies, and midwifery-led models of care (n=14), but also increasingly autonomous practice (n=9), implementing full scope of practice (n=8) and expanding the scope of midwifery (n=11), e.g. as experts in public health or reproductive health.

**Table 4:** Anticipated future changes in midwifery

*Subject*	*Number of mentions*
**Developing midwifery practice**	**32**
Midwifery-led models of care	14
Increasing autonomous practice	9
Implementing full scope of practice	8
Establishing the profession in practice	1
**Improving workforce conditions**	**17**
Protection of workforce, working conditions	6
Advocacy, recognition, promotion of midwifery	3
Regulation and certification	3
Strengthening the profession, leadership	3
Decreased finances, hospital demands, lack of midwives	2
**Expanding the scope of midwifery**	**11**
Expanding midwifery practice	7
Developing advanced midwifery roles, Advanced Midwifery Practise	4
**Improving politics for midwifery**	**8**
Changing legislation	3
Recognition of association	2
More political influence, midwife in government	3
**Improving midwifery education**	**6**
**Changes in health care, public health**	**5**
Humanising care and increased de-medicalisation	2
Increased user demands	1
Decreasing differences in interventions, equality in health	1
Increased digitalisation	1
**Developing midwifery research**	**4**

Another important theme was improvement of workforce issues and conditions (n=17). From the participants’ views, this involved protection of the workforce, improving workplace conditions and looking after the workforce in a time of increased user demand and economic constraints in health care organisations (n=8). This also meant, in particular, promotion and advocacy of midwifery as well as strengthening and regulating the profession (n=9) in order to move the professionalisation of midwifery forward.

## DISCUSSION

The aim of this study was to explore the current state of professionalisation of midwifery in Europe. The responses from participants evidence their perception that progress has been made with regard to developing a unique body of knowledge. In most countries, midwifery education has moved into institutes of higher education. The Bologna Declaration^12^ was instrumental in bringing about this change. However, some countries, such as Luxembourg and Germany, still have not been able to comply with this process^14^. More in-depth study on knowledge of barriers and facilitators of this process is therefore required.

Almost all countries provided opportunities for midwives’ academic postgraduate education. However, Master’s or doctoral studies are often offered in another subject or as an interdisciplinary study. While postgraduate education aims to provide more in-depth knowledge, and thus allows specialisation in one’s own professional field^4^, this might be viewed as a weakness, and ultimately change the character of the profession. A trend to incorporate postgraduate midwifery studies into nursing, as a specialisation within nursing has been noticed^6^. The small numbers of postgraduate students therefore require intense collaboration in national and international alliances^18^. In addition, the establishment of an international body of academically developed midwives, representing all fields of midwifery, would be an important means to bring professionalisation in Europe forward. As a consequence of an increased number of midwives undertaking postgraduate academic education, progress in midwifery research was also clearly outlined. The degree to which this was achieved, however, varied throughout Europe according to language (English-speaking or not English- speaking) and settings (university or practice). The current study indicated insufficient transfer of research findings into midwifery practice, although this varied regionally within countries. Two important influencing factors were highlighted: Firstly, the lack of clinical academic midwives, their recognition and influence, in maternity care practice in many countries^34^. This might be linked to the chosen model of academisation, which differs from the medical model. Secondly, the current position of midwives and their work in practice is an important factor.

The dissatisfaction of many countries’ delegates with their autonomy and current scope of midwifery practice highlighted the lack of progress, or even a backlash, in regard to the authority recognised by its clientele. As a matter of concern, midwives practised in line with the EU directives in most countries, but also reported an increase in obstetrician-led maternity care and the involvement of nurses in low-risk antenatal, intrapartum and postnatal care. Participants had little to say with regard to staffing, working conditions, and culture. Kirkham^30^ highlights the contrasting philosophies of current health care, and their effect on midwives, their competencies and philosophy of care. Some others reported on the negative effects of current working conditions on midwives that might make them leave the profession^35-37^. These contrasts were also found in this study. Sustainable professional autonomy is currently only achieved through independent midwife-led models of care. The importance of overarching European legislation, like the EU directives and cross-national collaboration was therefore emphasised. In line with this, future endeavours had to focus on (re-) establishing the full scope of midwifery practice, increased implementation of midwife-led models including continuity of care/carer, while expanding midwives’ roles and recognition in reproductive public health.

Insufficient or complete lack of progress was also noted with regard to broader community sanctions, code of ethics and a professional culture sustained by a professional organisation. National legislation and regulation of practice varied. Leadership in health service delivery and education, and participation in health policy, was still far from what would be expected, with midwives holding only few political decision-making positions throughout Europe. The current division of responsibility for the workforce in most European countries between the Departments of Health and Education weakens the profession as well.

The existence of codes of ethics for midwives in the majority of respondent countries was reassuring. Midwifery, however, cannot become a truly independent and autonomous profession simply with such structures being put into place. Codes of ethics, for example, do not mean that ethical practices will be followed, rather it is only by the inculturation of such codes into everyday practice that this will happen. Sustaining such a professional culture is viewed as a responsibility of the professional association^2^. Whereas national midwifery associations exist in all countries, some are a part of a larger nursing organisation. Advocating a unique culture and philosophy of care, while being partners with, or part of, other professional groups with different roots, poses big professional challenges for the future.

### Strengths and limitations

This was the first study to explore the current state of professionalisation of midwifery in Europe. The collection of new information on midwifery in 29 European countries enabled the defining of strengths and weaknesses, as well as opportunities and challenges while focusing on continuing the development of the profession. Limitations however included having key sources of information identified through EMA solely, which has the potential for misreporting of information, being based only on these expert opinions, as well as the questionnaire not being tested in a wider non-English speaking population. The latter was mediated through the option to contact the key person providing information for discussion. The acquired information, however, facilitated an evaluation of two decades of professionalisation of midwifery in Europe, with a view of progress or failure to progress.

## CONCLUSIONS

International recognition of the midwifery contribution to improve maternal and infant health has been clearly stated, and is widely demonstrated by evidence^38^. To be autonomous partners in maternity care, and a valuable resource for achieving optimum results, midwives need a strong profession in all countries in Europe. This study shows progress in the areas of education and academic research; however, it also highlights serious weaknesses and challenges with regard to midwives’ current roles in maternity care practice and legislation, as well as their influence on health care culture and politics. Advancing professionalisation should therefore focus on three main areas (Table 5). First, securing, monitoring and advancing (the scope of) midwifery practice, in which more in-depth studies on midwives’ work, conditions and culture are needed. Second, considering (re)defining the profession in the larger area of reproductive public health, to be and maintain professional experts for the health of women and families in Europe. Third, in order to achieve these aims, there is a need to define a pan-European strategy for roles for midwives with postgraduate degrees, in all fields of midwifery. The latter should serve as an essential resource, and implementation to include lobbying for strengthening professional political leadership and development. The WHO recommendations for strengthening the midwifery profession and encouraging their participation in all levels of decision underpin these efforts^39^. National and regional governments have to take all this in consideration when regulating and planning health services, which includes allocating the appropriate resources in the right positions. A strong midwifery profession is needed to achieve optimal results in terms of health and costs, not only for the health system but also for the whole society.

**Table 5:** Recommendations for advancing the professionalisation of midwifery in Europe

Secure, monitor and advance (the scope of) midwifery practice, including in-depth studies on midwives’ work, conditions and culture
Re-define the profession in the larger area of reproductive public health, to be, and maintain professional experts for, the health of women and families in Europe
Define a pan-European strategy for roles for midwives with postgraduate degrees for all fields of midwifery

## References

[1] Kethler U (1998a). Professionalisierung - ein relevanter Weg für Hebammen!. Deutsche Hebammenzeitschrift.

[2] Zoege M (2004). Die Professionalisierung des Hebammenberufs: Anforderungen an die Ausbildung.

[3] Keogh J (1997). Professionalization of nursing: development, difficulties and solutions. J Adv Nurs.

[4] Gerrish K, McManus M, Ashworth P (2003). Creating what sort of professional? Master's level nurse education as a professionalising strategy. Nurs Inq.

[5] Larkin G (1995). Health professions and the state in Europe.

[6] Manzano-García G, Ayala-Calvo JC (2014). An overview of nursing in Europe: a SWOT analysis. Nurs Inq.

[7] Hamilton PM (1996). Realities of contemporary nursing.

[8] Kethler U (1998b). Professionalisierung- ein relevanter Weg für Hebammen!. Deutsche Hebammenzeitschrift.

[9] Mander R, Fleming V (2002). Failure to progress: the contraction of the midwifery profession.

[10] World Health Organization (2015). European strategic directions for strengthening nursing and midwifery towards Health 2020 goals.

[11] (2013). Directive 2013/55/EU of the European Parliament and of the Council: of 20th November 2013, amending Directive 2005/36/EC on the recognition of professional qualifications and Regulation (EU) No 1024/2012 on administrative cooperation through the Internal Market Information System. ficial Journal of the European Union.

[12] European Ministers in charge of Higher Education The Bologna Declaration of 19 June 1999: Joint declaration of the European Ministers of Education.

[13] Van der Wende MC (2000). The Bologna Declaration: Enhancing the transparency and competitiveness of European higher education. Journal of Studies in International Education.

[14] Plappert C, Graf J, Simoes E, Schönhardt S, Abele H (2019). The Academization of Midwifery in the Context of the Amendment of the German Midwifery Law: Current Developments and Challenges. Geburtshilfe und Frauenheilkunde.

[15] Vermeulen J, Luyben A, Jokinen M, Matintupa E, O'Connell R, Bick D (2018). Establishing a Europe-wide foundation for high quality midwifery education: The role of the European Midwives Association (EMA). Midwifery.

[16] Fleming V, Pehlke-Milde J, Davies S, Zaksek T (2011). Developing and validating scenarios to compare midwives’ knowledge and skills with the International Confederation of Midwives’ essential competencies in four European countries. Midwifery.

[17] Praxmarer-Fernande S, Maier CB, Oikarainen A, Buchan J, Perfilieva G, Organization WH (2017). Levels of education offered in nursing and midwifery education in the WHO European region: multicountry baseline assessment. Public Health Panorama.

[18] Fleming V, Luyben A (2016). Establishing a Master's for Europe-A transnational model for higher education. Midwifery.

[19] EU Executive Agency for Health and Consumers (2015). Study Concerning the Review and Mapping of Continuous Professional Development and Life- long Learning for Health Professionals in the EU: Final Report.

[20] World Health Organization (1985). Having a baby in Europe: Public Health Europe 26.

[21] Kahl CM (2013). Stand der Entwicklung der Hebammenwissenschaft-Beschreibung der Ist-Situation anhand der Diskursuntersuchung der Forschungslage.

[22] Robinson S, Thomson A (1993). Midwives, research and childbirth.

[23] Luyben A, Wijnen H, Oblasser C, Perrenoud P, Gross M (2013). The current state of midwifery and development of midwifery research in four European countries. Midwifery.

[24] Goyet S, Sauvegrain P, Schantz C, Morin C (2018). State of midwifery research in France. Midwifery.

[25] Clesse C, Lighezzolo-Alnot J, de Lavergne S, Hamlin S, Scheffler M (2018). The evolution of birth medicalisation: A systematic review. Midwifery.

[26] Feeley C (2019). The medicalisation of women’s bodies. The practising Midwife.

[27] Van Teijlingen E (2005). A critical analysis of the medical model as used in the study of pregnancy and childbirth. Sociological Research Online.

[28] Brailey S, Luyben A, Van Teijlingen E, Frith L (2017). Women, Midwives, and a Medical Model of Maternity Care in Switzerland. International Journal of Childbirth.

[29] Prosen M, Krajnc MT (2019). Perspectives and experiences of healthcare professionals regarding the medicalisation of pregnancy and childbirth. Women Birth.

[30] Kirkham M (2019). Midwives should…. Birth Practice and Politics Forum.

[31] Edwards N, Mander R, Murphy-Lawless J (2018). Untangling the Maternity Crisis.

[32] Creswel JW (2009). Research design: Qualitative, quantitative, and mixed methods approaches.

[33] Braun V, Clarke V (2006). Using thematic analysis in psychology. Qualitative Research in Psychology.

[34] Luyben A, Barger MK, Avery MD, Bick D (2018). What is next? Midwifery education building partnerships for tomorrow's maternal and neonatal health care. Midwifery.

[35] Beier L (2016). Was brauchen Hebammen in Österreich und der Schweiz, damit sie im Beruf bleiben? Masterthesis MSc Advanced Midwifery Practice.

[36] Hunter B, Fenwick J, Sidebotham M, Henley J (2019). Midwives in the United Kingdom: Levels of burnout, depression, anxiety and stress and associated predictors. Midwifery.

[37] O’Riordan S, O'Donoghue K, McNamara K (2019). Interventions to improve wellbeing among obstetricians and midwives at Cork University Maternity Hospital. Irish Journal of Medical Science (1971-).

[38] Renfrew MJ, Homer C, Downe S (2014). Midwifery: an executive summary for The Lancet’s series. Lancet.

[39] Buscher A, Sivertsen B, White J (2010). Nurses and Midwives: A Force for health: Survey on the situation of nursing and midwifery in the Member States of the European Region of the World Health Organization 2009.

